# Determination of Human Papillomavirus Type 18 Lineage of E6: A Population Study from Iran

**DOI:** 10.1155/2022/2839708

**Published:** 2022-03-18

**Authors:** Mona Sadat Larijani, Mir Davood Omrani, Rahim Soleimani, Anahita Bavand, Amir Houshang Nejadeh, Vahid Ezzatizadeh, Mahboubeh Jamshidi, Amitis Ramezani

**Affiliations:** ^1^Clinical Research Department, Pasteur Institute of Iran, Tehran, Iran; ^2^Department of Medical Genetics, Shahid Beheshti University of Medical Sciences, Tehran, Iran; ^3^Department of Virology, School of Public Health, Tehran University of Medical Sciences, Tehran, Iran; ^4^Ayandeh Clinical and Genetic Laboratory, Varamin, Iran

## Abstract

The epidemiological studies in Iran on HPV18 nucleotide changes are rare. This type of virus is prevalent in the Iranian population. Therefore, in the present study, we aimed to identify the genetic variability in HPV18 in the E6 region to evaluate the prevalence of lineage distribution and sublineages in a sample population in Iran. Overall, 60 HPV18 confirmed cases were investigated between 2019 and 2021. The specimens were collected, and molecular genotyping was done using the Linear Array HPV Genotyping Test. DNA extraction was performed by a viral DNA/RNA kit. The HPV E6 gene was amplified by using type-specific primers designed according to the HPV18 genome prototype sequence. The sequencing of the E6 region was successfully done on 43 samples which were then compared to the reference sequence. The most frequent sublineage of HPV18 in this study was A4 (69.7%), followed by A1 (18.6%) and A3 (11.6%). Neither A2 nor A5 sublineage was not detected in this study. The related nucleotide acid changes according to the main references were as follows: A3: T104C/T232G/T485C/C549A, A4: T104C/T485C/C549A. The predominance of A lineage with the high frequency of A4 sublineage was found in the present research. The importance of sublineages in susceptibility to a progressive form of infection requires to be more investigated among the different population.

## 1. Introduction

Human papillomavirus (HPV) is a well-known cause of cervical intraepithelial neoplasia (CIN) and invasive cervical cancer (CC) as the third most prevalent cancer among females [1, 2]. Generally, HPV epidemiologic distribution and its associated burden are significantly different in the world, and the mortality rate is affected by viral genome variability as well as host's factors including age and health state [3].

There have been more than 200 identified HPV types from which 13-15 kinds are carcinogenic. Among the high-risk genotypes, HPV16 and HPV18 are considered the most common oncogenic HPV genotypes causing approximately 70% of all cervical cancers [4, 5].

The contribution of HPV genotypes to cervical cancer differs across the world which may stem from geographic variations in HPV type-specific prevalence among the populations. Cervix HPV infections are mostly asymptomatic, and about 90% of detected infections are cleared in two years although the protection and duration of immunity after a natural infection has remained unknown [6–8].

According to the published data, the highest HPV prevalence was found in Oceania, Africa, Europe, America, and Asia, respectively [2]. The four most frequent HPV types in the Iranian population were shown to be HPV16 and HPV18 followed by types 66 and 11 which represent 63.8% of all HPV infections [9]. This is consistent with conducted most studies in the rest of the world where HPV16 has been reported as the most frequent type, followed by HPV18 [9, 10].

In the other study which included 1218 Iranian samples with normal cervical cytology, HPV was found in 7.2% which varied according to the geographical regions. The data showed HPV was most common in the North (11.7%) whereas the lowest in the Center (4.5%) of Iran [9].

HPV variants have different phenotypic characteristics in terms of carcinogenicity and cancer progression. In addition to the virus type, there have been some reported substitutions in oncogenes which might affect the related protein function and thus the infection progress as well [11].

HPV18 has been classified into three main variants such as European, African, and Asian-Amerindian variants [12]. HPV variants of a type share >90% nucleotide identities. Therefore, variant lineages differ by approximately 1–10% whereas sublineages include 0.5-1% differences among a given type [13].

Genetic variation has been shown to be associated with different manifestations of infectious diseases even in the host or the pathogen [14–17]. Therefore, how human/pathogen genetics influence the disease progress or susceptibility to it brings the opportunity for new insights into diagnosis, potential medicine targets, risk factors, vaccination, and response to therapy [18–21]. HPV E6 and E7 as the major oncogenes are highly expressed in tumors and are associated with cellular transformation, immortalization, and carcinogenesis via protein–protein interactions with tumor suppressor proteins like p53 and Rb [22].

Investigation on HPV variants and also probable nucleotide variability is essential to assess the nucleotide variations which may contribute to the viral oncogenic potential. Moreover, the host cellular immune response might be affected by amino acid substitutions on the viral capsid. Finally, HPV infection of a particular variant may not raise immunological protection against another subsequent incidence of the other variant with the same genotype [23].

The epidemiological studies in Iran, which refer to HPV18 nucleotide variability as a frequent type in the region, are rare. Therefore, in the present study, we aimed to characterize the genetic variability of HPV18 regarding the E6 genomic region to assess the prevalence of HPV18 lineage and sublineages in a sample population of our country.

## 2. Materials and Methods

### 2.1. Sample Collection

60 cervical samples were collected from cases referred to medical centers from 2019 to 2021 for routine screening. Molecular genotyping was achieved using the “Linear Array HPV Genotyping Test” kit (Roche Molecular Diagnostics). Then, HPV18-diagnosed samples were excluded and applied for further steps. All the procedures were approved by National Institute for Medical Research Development (IR.NIMAD.REC.1398.331) and complied with the guidelines and ethical standards for experimental investigation with human subjects of the Helsinki Declaration.

### 2.2. Genomic DNA Extraction

DNA extraction was performed by the viral DNA/RNA kit (FAVORGEN Biotech, Taiwan) according to the manufacturer's instructions. Extracted DNA was eluted with 50 *μ*l AE buffer and stored at -20°C until amplification.

### 2.3. PCR Amplification and Sequencing

Amplification of HPV E6 was performed using type-specific primers designed according to the HPV18 genome prototype sequence (GenBank accession number NC001357). PCR was performed in 25 *μ*l of reaction mixture containing 10X PCR buffer, 25 mmol/l MgCl_2_, 25 mmol/l of each dNTP, 100 pmol/l of sense and anti-sense primer, 5 *μ*l of template DNA, and 2.5 U of Taq DNA polymerase (Yekta Tajhiz, Iran). The initial denaturation started at 95°C for 5 min, followed by 42 cycles of 53°C annealing temperature, and finished with a final extension at 72°C for 5 min (Table 1). PCR products were detected in the agarose gel (1%). The sequences were subsequently analyzed (sequencer technology information) by the Virology Department of Tehran University and then were compared to the reference sequence of the E6 gene in order to identify gene polymorphisms using bioinformatics tool.

The same forward-specific primers applied in amplification were then used as the sequencing primers. HPV obtained sequences were aligned in NCBI and compared to the reference HPV18-type sequence which belongs to the Asian-Amerindian lineage (accession number NC001357) by the MEGA 6.0 tool.

### 2.4. Statistical Analysis

The SPSS 26 program for statistical analysis was applied, and a *p* value less than 0.05 was considered significant.

## 3. Results

**Figure 1:**
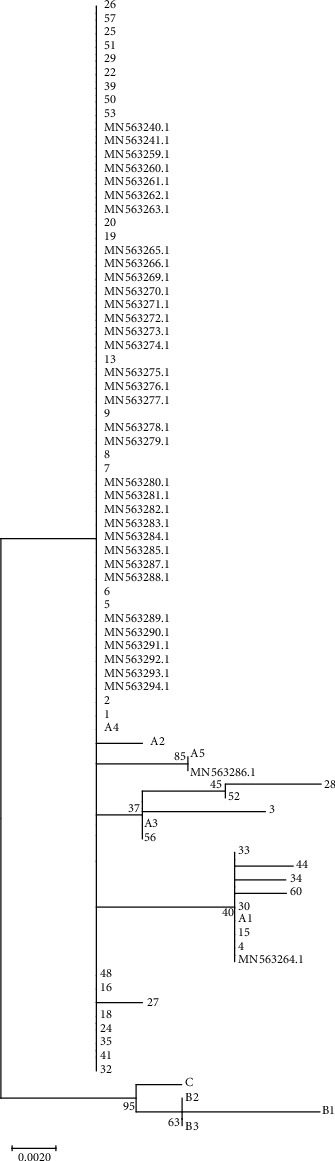
Phylogenetic analysis of HPV 18 E6 gene was carried out by the MEGA6 software using the maximum likelihood method based on the Kimura 2‐parameter model. The accession number of reference sequences used in this study were as follows: AY262282, A1; EF202146, A2; EF202147, A3; EF202151, A4; GQ180787, A5; EF202155, B1; KC470225, B2; EF202152, B3; and KC470229, C, as indicated by letters A1-A5. MN563240.1-MN563294.1 sequences were obtained from the study by Salavatiha et al. (28), and the sequenced data of this study are mentioned by cardinals.

In this study, 60 women aged 21-49 (mean: 34.26) who were referred to the medical centers for routine screening were enrolled. Overall, 43 collected cervical samples were of proper quality for further steps. E6 fragment was amplified and subjected to sequencing. The sequencing analysis by the MEGA 6.0 tool showed that all the samples belonged to lineage A of HPV18 which is common in Iran (Figure 1). The related sublineages in this study are shown in Table 2. The frequency of HPV18 sublineages was A4 (69.7%), followed by A1 (18.6%) and A3 (11.6%). Neither A5 nor A2 was not observed in any cases. The related nucleotide acid changes according to the main references were as follows: A3: T104C/T232G/T485C/C549A, A4: T104C/T485C/C549A. Moreover, the cytology assessment profile of the cases showed that the association between CIN and these variants was not statistically significant (*p* < 0.05).

## 4. Discussion

HPV18 variants require to be more investigated in Iran regardless of the fact that HPV18 prevalence is common in the population. This genotype besides genotype 16 causes 70% of cervical cancer cases which highlights the importance of the epidemiologic studies and determination of the virus variations.

The distribution of HPV variants has been found to be associated with race or geographic region [24, 25]. This fact may also affect the oncogenic potency among variants. HPV18 has been considered to have three lineages: A (previously called Asian-American and European lineages (AA and E respectively)) and B and C (these two include previous African (Af)). In addition, eight sublineages have been classified for this genotype [13].

The predominance of A variant (including Asian-Amerindian and European) is supposed for the Persian population which is in accordance with the result of this study. Non-European HPV18 variants have been found to be associated with preinvasive lesions and are also more persistent [26]. In a study by Hecht et al., an HPV variant was present in 40% of intraepithelial lesions although it had less oncogenic risk [27]. Moreover, most studies confirmed that different variants belonged to the same genotype that may have different pathogenic characteristics, and thus, nucleotide substitutions can play a crucial role.

In the previous study in Iran, lineage A was detected in all collected samples, and similar to our finding, sublineage A4 was the most frequent type [28]. Lineage A of HPV18 was predominant in Spain which is similar to this study. What is more, they found that lineage B of HPV18 can be associated with a high risk of cervical cancer in comparison with lineage A [29]. HPV18 studied genomes in China were assigned to the A3/A4 and A1 sublineages harboring 73 variations (0.93% of the genome), including 1 insertion, 71 substitutions, and 1 deletion [30].

In the other investigation on 25 Taiwanese with HPV18 cases, 7 of them were coinfected with HPV16. C183G of the E6 as a silence mutation was the most dominant mutation among all the subjects [31].

In the other study in Spain, Basaras et al. found that specific African substitutions were related to the high-grade squamous intraepithelial lesion samples. They concluded that non-European HPV variant prevalence is a risk factor for persistence and progression [23].

It was determined that mutations in the last nucleotide of the LCR, at position 104, modified Sp1 and YY1 activity, and this variation is related to the higher activity of the E6/E7 promoter. We determined T104C mutation in most of our cases (71.5%). Interestingly, cancer recurrence is less likely to occur in women with T104C mutations in contrast to those infected with wild-type [23].

Shin et al. determined that all the investigated samples from Korean HPV18-positive patients belonged to linage A. Moreover, sublineage A1 comprised 91.7%. Fifteen new nucleotide substitutions were identified among 44 nucleotide substitutions from which six substitutions at positions 317, 443, 5467, 5560, 6462, and 6823 resulted in amino acid changes in E6 including F71L, N113K; L1: H13R, H44P, A345T, and N465S [32]. Among the reference sublineages, A3 was reported to harbor amino acid changes from E to G at position 43 in contrast to other HPV18 sublineages. We detected E43G amino acid change in the A3 sublineage in five cases, and other nucleotide changes did not lead to varied amino acids. A Chinese study also studied 122 HPV18 women from 2012 to 2015. The sequencing of E6 and E7 showed that all HPV18 belonged to lineage A while no lineage B was seen which is similar to our findings. Moreover, A1 was the most common variant (85.2%) followed by sublineages A4, A3, and A5, with no sublineage A2. Nevertheless, A4 was the dominant sublineage in Iran and no A5 was found [23].

## 5. Conclusion

In the present study, lineage A was identified in Iranian females with HPV18. So far, the A4 sublineage of HPV18 is the most prominent variant of HPV18 in Iran whereas neither A2 nor A5 was not detectable. The gathering data of HPV18 distribution and geographic diversity provides us with critical information to expand diagnostic tools and epidemiologic association with cervical cancer risk and also to come up with a proper HPV vaccine type for targeted populations worldwide.

## Figures and Tables

**Table 1:** Polymerase chain reaction characteristics for the E6 gene of HPV18.

	Primer sequence (5′–3′)	Annealing temp/cycles	Amplified nucleotides (E6 region is 105-581/primer target is 80-746)	Amplicon length
E6 F	GATGTGAGAAACRCACCACCA	53°C/42 rep	105-581	667 bp
E6 R	GTCGGGCTGGTAAATGTTGAT			

**Table 2:** HPV 18 sublineages identified in Iranian women.

Sublineage	Acid nucleic substitution	Amino acid changes	Nucleotide position									Total (%)
			104^∗^	149	232	377	442	485	523	549	570	*N* = 43
A1	Prototype	-	T	A	T	A	A	T	C	C	A	8 (18.6)
A3	T104C^∗^/T232G/T485C/C549A	E43G	C	A	G	A	A	C	C	A	A	5 (11.6)
A4	T104C^∗^/T485C/C549A	-	C	A	G	A	A	C	C	A	A	30 (69.7%)
A5	T104C^∗^/A149G/A377G/T485C/C549A	-	C	C	G	G	A	C	C	A	A	-

Note: ^∗^Nucleotide of 104 is located at the end of the long control region of HPV 18.

## Data Availability

The data that support the findings of this study are available from the corresponding author upon reasonable request.
